# Supplementary material for: Estimating the prevalence and characteristics of people in severe social isolation in 29 European countries: A secondary analysis of data from the European Social Survey round 9 (2018–2020)
Source: PLoS One. 2023 Sep 12;18(9):e0291341. doi: 10.1371/journal.pone.0291341 (PMC10497126; doi:10.1371/journal.pone.0291341)
Supplement: S1 Table — (DOCX) [file pone.0291341.s001.docx]

**S1 Table. List of indicators used in this study.**

| **Acronym** | **Variable** | **Value/Category** |
| --- | --- | --- |
|  | **Severe social isolation with high risk of hikikomori** |  |
| sclmeet | How often do you meet socially with friends, relatives or work colleagues? | Never, Less than once a month |
| Sclact | Compared to other people of your age, how often would you say you take part in social activities? | Much less than most, Less than most |
| Pdwrk | In paid work (or away temporarily) (employee, self-employed, working for your family business) during the last 7 days | Not marked |
| uempla | Unemployed, actively looking for job during the last 7 days | Not marked |
| Edctn | In education, (not paid for by employer) even if on vacation during the last 7 days | Not marked |
|  | **Sociodemographic characteristics** |  |
| Gndr | Sex | 1 = Male, 2 = Female |
| Agea | Age of respondent | Ranging from 15 to 64 |
| hhmmb | Including yourself, how many people – including children – live here regularly as members of this household? | In number |
| livealone | Respondent lives alone | 0 = No, 1 = Yes |
| livewhusband | Respondent lives with husband/wife/partner | 0 = No, 1 = Yes |
| livewparent | Respondent lives with at least one parent, parent-in-law, partner’s parent, step parent | 0 = No, 1 = Yes |
| Bthcld | Have you ever given birth to/fathered a child? | 0 = No, 1 = Yes |
| domicil | Which phrase on this card best describes the area where you live? | 1 = A big city, 2 = The suburbs or outskirts of a big city, 3 = A town or a small city, 4 = A country village, 5 = A farm or home in the countryside |
| cntry | European country |  |
| region | Region of Europe according to EuroVoc | 1 = Northern (Denmark, Estonia, Finland, Iceland, Latvia, Lithuania, Norway, Sweden), 2 = Southern (Cyprus, Italy, Portugal, Spain), 3 = Western (Austria, Belgium, France, Germany, Ireland, Netherlands, Switzerland, United Kingdom), 4 = Central and Eastern Europe (Bulgaria, Czech Republic, Croatia, Hungary, Montenegro, Poland, Serbia, Slovakia, Slovenia) |
| eisced | What is the highest level of education you have successfully completed? | From 1 = ES-ISCED I, less than lower secondary to 7 = ES-ISCED V2, higher tertiary education, >= MA level |
| eduyrs | About how many years of education have you completed, whether full-time or part-time? Please report these in full-time equivalents and include compulsory years of schooling | In number |
| dsbld | Permanently sick or disabled | 0 = No, 1 = Yes |
| hlthhmp | Are you hampered in your daily activities in any way by any longstanding illness, or disability, infirmity or mental health problem? | 0 = No, 1 = Yes |
| hswrk | Doing housework, looking after children or other persons | 0 = No, 1 = Yes |
| rtrd | Retired | 0 = No, 1 = Yes |
| netusoft | People can use the internet on different devices such as computers, tablets and smartphones. How often do you use the internet on these or any other devices, whether for work or personal use? | From 1 = Never to 5 = Every day |
| brncntr | Born in country | 0 = Yes, 1 = Other country |
| facntr, mocntr | Father/Mother born in country | 0 = Yes, 1 = At least one born in other country |
| eiscedf | What is the highest level of education your father successfully completed? | From 1 = ES-ISCED I, less than lower secondary to 7 = ES-ISCED V2, higher tertiary education, >= MA level |
| emprf14 | When you were 14, did your father work as an employee, was he self-employed, or was she not working then? | 0 = (Self-/)Employed, 1 = Unemployed, 2 = Father dead/absent |
| eiscedm | What is the highest level of education your mother successfully completed? | From 1 = ES-ISCED I, less than lower secondary to 7 = ES-ISCED V2, higher tertiary education, >= MA level |
| emprm14 | When you were 14, did your mother work as an employee, was she self-employed, or was she not working then? | 0 = (Self-/)Employed, 1 = Unemployed, 2 = Mother dead/absent |
| hincsrca | Please consider the income of all household members and any income which may be received by the household as a whole. What is the main source of income in your household? | 1 = Wages or salaries/ Income from self-employment (excluding farming)/ Income from farming/ Pensions, 2 = Unemployment/redundancy benefit, 3 = Any other social benefits or grants, 4 = Income from investments, savings etc./ Income from other sources |
| hinctnta | Household's total net income, all sources | In deciles |
| hincfel | Which of the descriptions comes closest to how you feel about your household’s income nowadays? | 1 = Living comfortably on present income, 2 = Coping on present income, 3 = Finding it difficult on present income, 4 =Finding it very difficult on present income |
| iincsrc | Now please consider your own individual income. What is your main source of income? | 1 = Wages or salaries/ Income from self-employment (excluding farming)/ Income from farming/ Pensions, 2 = Unemployment/redundancy benefit, 3 = Any other social benefits or grants, 4 = Income from investments, savings etc./ Income from other sources, 5 = No personal income |
|  | **Adverse events and fear** |  |
| crmvct | Have you or a member of your household been the victim of a burglary or assault in the last 5 years? | 0 = No, 1 = Yes |
| aesfdrk | How safe do you – or would you – feel walking alone in this area after dark? Do – or would – you feel… | From 1 = very safe to 4 = very unsafe |
|  | **Social support and trust** |  |
| inprdsc | How many people, if any, are there with whom you can discuss intimate and personal matters? | From 0 = None to 6 = 10 or more |
| ppltrst, pplfair, pplhlp | Social trust scale (Breyer, 2015): Most people can be trusted or you can't be too careful, Most people try to take advantage of you, or try to be fair, Most of the time people helpful or mostly looking out for themselves | From 0 = Low trust to 10 = High trust |
| trstprl, trstlgl, trstplc, trstplt, trstprt | Political trust (Besic 2021): Trust in country's parliament, Trust in the legal system, Trust in the police, Trust in politicians, Trust in political parties | From 0 = No trust at all to 10 = Complete trust |
|  | **Well-being** |  |
| stflife | All things considered, how satisfied are you with your life as a whole nowadays? | From 0 = Extremely dissatisfied to 10 = Extremely satisfied |
| happy | Taking all things together, how happy would you say you are? | From 0 = Extremely unhappy to 10 = Extremely happy |
| health | How is your health in general? Would you say it is… | From 1 = Very good to 5 = Very bad |
| plnftr | Do you generally plan for your future or do you just take each day as it comes? | From 0 = I plan for my future as much as possible to 10 = I just take each day as it comes |
