# Supplementary material for: Estimating the prevalence and characteristics of people in severe social isolation in 29 European countries: A secondary analysis of data from the European Social Survey round 9 (2018–2020)
Source: PLoS One. 2023 Sep 12;18(9):e0291341. doi: 10.1371/journal.pone.0291341 (PMC10497126; doi:10.1371/journal.pone.0291341)
Supplement: S2 Table — SE: Standard error, CI: Confidence intervals. (DOCX) [file pone.0291341.s002.docx]

**S2 Table. Severe social isolation prevalence according to rounds 1, 9 and 10 of the European Social Survey (ESS) for ten countries with available data in each survey (Czech Republic, Finland, France, Hungary, Italy, Netherlands, Norway, Portugal, Slovenia, Switzerland).**

|  | **ESS round (years)** | **Sex** | **Population total** | **Population %** | **Weighted count** | **Weighted % (SE)** | **Weighted 95% C.I.** |
| --- | --- | --- | --- | --- | --- | --- | --- |
| Severe social isolation | 1  (2002-03) | Total | 317 | 2.34 | 278.56 | 2.31 (0.24) | 1.84, 2.78 |
|  |  | Male | 147 | 2.32 | 118.09 | 2.14 (0.31) | 1.53, 2.75 |
|  |  | Female | 170 | 2.36 | 160.48 | 2.46 (0.35) | 1.78, 3.15 |
| Severe social isolation | 9  (2018-20) | Total | 202 | 1.58 | 190.00 | 1.55 (0.19) | 1.17, 1.92 |
|  |  | Male | 71 | 1.16 | 71.97 | 1.19 (0.25) | 0.70, 1.67 |
|  |  | Female | 131 | 1.97 | 118.04 | 1.91 (0.28) | 1.36, 2.45 |
| Severe social isolation | 10  (2020-22) | Total | 230 | 1.76 | 187.55 | 1.52 (0.15) | 1.22, 1.82 |
|  |  | Male | 65 | 1.05 | 62.72 | 1.03 (0.17) | 0.69, 1.37 |
|  |  | Female | 165 | 2.49 | 124.83 | 2.00 (0.24) | 1.53, 2.46 |

*SE:* standard error, *CI:* confidence intervals.
