# Supplementary material for: Estimating the prevalence and characteristics of people in severe social isolation in 29 European countries: A secondary analysis of data from the European Social Survey round 9 (2018–2020)
Source: PLoS One. 2023 Sep 12;18(9):e0291341. doi: 10.1371/journal.pone.0291341 (PMC10497126; doi:10.1371/journal.pone.0291341)
Supplement: S3 Table — CI: Confidence intervals. (DOCX) [file pone.0291341.s003.docx]

**S3 Table. Severe social isolation prevalence according to country (European Social Survey round 9).**

| **Country** | **Weighted %** | **Weighted 95% C.I.** |
| --- | --- | --- |
| Austria | 1.16 | 0.61, 1.72 |
| Belgium | 1.44 | 0.8, 2.09 |
| Bulgaria | 3.26 | 2.32, 4.21 |
| Croatia | 2.11 | 1.2, 3.02 |
| Cyprus | 3.48 | 1.76, 5.21 |
| Czech Republic | 1.41 | 0.83, 1.99 |
| Denmark | 0.77 | 0.16, 1.38 |
| Estonia | 2.39 | 1.6, 3.18 |
| Finland | 0.72 | 0.24, 1.2 |
| France | 1.09 | 0.25, 1.93 |
| Germany | 1.5 | 0.78, 2.22 |
| Hungary | 5.79 | 4.32, 7.26 |
| Iceland | 0.2 | -0.19, 0.59 |
| Ireland | 3.29 | 2.31, 4.28 |
| Italy | 2.02 | 1.28, 2.76 |
| Latvia | 0.96 | 0.3, 1.63 |
| Lithuania | 3.05 | 1.85, 4.25 |
| Montenegro | 0.53 | 0.15, 0.9 |
| Netherlands | 0.85 | 0.31, 1.39 |
| Norway | 0.2 | -0.19, 0.59 |
| Poland | 2.87 | 1.87, 3.86 |
| Portugal | 1.15 | 0.42, 1.88 |
| Serbia | 1.88 | 1.12, 2.64 |
| Slovakia | 2.34 | 1.1, 3.59 |
| Slovenia | 2 | 1.14, 2.86 |
| Spain | 0.89 | 0.37, 1.42 |
| Sweden | 0.41 | 0.03, 0.79 |
| Switzerland | 0.41 | 0.05, 0.78 |
| United Kingdom | 2.85 | 1.98, 3.71 |

*CI:* confidence intervals.
