# Supplementary material for: Estimating the prevalence and characteristics of people in severe social isolation in 29 European countries: A secondary analysis of data from the European Social Survey round 9 (2018–2020)
Source: PLoS One. 2023 Sep 12;18(9):e0291341. doi: 10.1371/journal.pone.0291341 (PMC10497126; doi:10.1371/journal.pone.0291341)
Supplement: S4 Table — CI: Confidence intervals. (DOCX) [file pone.0291341.s004.docx]

**S4 Table. Severe social isolation weighted prevalence according to rounds 1, 9 and 10 of the European Social Survey (ESS) for each of the ten countries with available data (Czech Republic, Finland, France, Hungary, Italy, Netherlands, Norway, Portugal, Slovenia, Switzerland).**

| **Country** | **ESS round (years)** | **Total** | |  | **Sex** | | | |
| --- | --- | --- | --- | --- | --- | --- | --- | --- |
|  |  |  |  |  | **Male** | | **Female** | |
|  |  | % | 95% C.I. |  | % | 95% C.I. | % | 95% C.I. |
| Czech Republic | 1 (2002-03) | 2.2 | 1.18, 3.22 |  | 2.17 | 0.9, 3.45 | 2.26 | 0.69, 3.83 |
|  | 9 (2018-20) | 1.41 | 0.83, 1.99 |  | 1.4 | 0.64, 2.15 | 1.42 | 0.53, 2.31 |
|  | 10 (2020-22) | 1.66 | 1.05, 2.27 |  | 0.67 | 0.07, 1.27 | 2.65 | 1.58, 3.73 |
| Finland | 1 (2002-03) | 0.82 | 0.38, 1.25 |  | 0.74 | 0.14, 1.35 | 0.89 | 0.26, 1.51 |
|  | 9 (2018-20) | 0.72 | 0.24, 1.2 |  | 0.18 | -0.17, 0.52 | 1.27 | 0.37, 2.16 |
|  | 10 (2020-22) | 1.27 | 0.43, 2.11 |  | 1.23 | -0.16, 2.63 | 1.31 | 0.38, 2.24 |
| France | 1 (2002-03) | 1.68 | 0.9, 2.46 |  | 1.43 | 0.26, 2.6 | 1.9 | 0.87, 2.93 |
|  | 9 (2018-20) | 1.09 | 0.25, 1.93 |  | 0.92 | -0.27, 2.1 | 1.26 | 0.07, 2.44 |
|  | 10 (2020-22) | 0.78 | 0.33, 1.23 |  | 0.93 | 0.22, 1.63 | 0.64 | 0.07, 1.21 |
| Hungary | 1 (2002-03) | 9.07 | 7.53, 10.62 |  | 9.91 | 7.35, 12.47 | 8.25 | 6.54, 9.97 |
|  | 9 (2018-20) | 5.79 | 4.32, 7.26 |  | 3.43 | 1.88, 4.98 | 8.11 | 5.66, 10.56 |
|  | 10 (2020-22) | 5.17 | 3.83, 6.52 |  | 3.13 | 1.48, 4.79 | 7.21 | 5.15, 9.27 |
| Italy | 1 (2002-03) | 2.87 | 1.7, 4.03 |  | 2.49 | 1.06, 3.93 | 3.16 | 1.42, 4.9 |
|  | 9 (2018-20) | 2.02 | 1.28, 2.76 |  | 1.37 | 0.55, 2.2 | 2.66 | 1.55, 3.77 |
|  | 10 (2020-22) | 2.17 | 1.4, 2.94 |  | 1.06 | 0.39, 1.73 | 3.25 | 1.96, 4.55 |
| Netherlands | 1 (2002-03) | 1 | 0.54, 1.45 |  | 0.99 | 0.33, 1.64 | 1.01 | 0.37, 1.65 |
|  | 9 (2018-20) | 0.85 | 0.31, 1.39 |  | 1.4 | 0.41, 2.38 | 0.32 | -0.13, 0.78 |
|  | 10 (2020-22) | 0.91 | 0.31, 1.52 |  | 0.7 | -0.09, 1.48 | 1.13 | 0.2, 2.06 |
| Norway | 1 (2002-03) | 0.45 | 0.11, 0.8 |  | 0.54 | 0, 1.08 | 0.37 | -0.05, 0.8 |
|  | 9 (2018-20) | 0.2 | -0.19, 0.59 |  | 0.38 | -0.37, 1.14 | 0 | 0, 0 |
|  | 10 (2020-22) | 1.31 | 0.2, 2.42 |  | 1.79 | -0.06, 3.64 | 0.81 | -0.37, 2 |
| Portugal | 1 (2002-03) | 0.78 | 0.25, 1.3 |  | 0.41 | -0.07, 0.9 | 1.07 | 0.21, 1.92 |
|  | 9 (2018-20) | 1.15 | 0.42, 1.88 |  | 0.78 | -0.17, 1.73 | 1.47 | 0.39, 2.56 |
|  | 10 (2020-22) | 1.42 | 0.72, 2.13 |  | 0.57 | 0.04, 1.11 | 2.2 | 0.94, 3.45 |
| Slovenia | 1 (2002-03) | 4.63 | 3.44, 5.82 |  | 3.26 | 1.89, 4.62 | 5.9 | 4.11, 7.7 |
|  | 9 (2018-20) | 2 | 1.14, 2.86 |  | 1.1 | 0.24, 1.95 | 2.97 | 1.54, 4.41 |
|  | 10 (2020-22) | 0.82 | 0.25, 1.39 |  | 0.56 | -0.08, 1.2 | 1.11 | 0.14, 2.08 |
| Switzerland | 1 (2002-03) | 0.19 | -0.03, 0.41 |  | 0.1 | -0.1, 0.3 | 0.28 | -0.11, 0.66 |
|  | 9 (2018-20) | 0.41 | 0.05, 0.78 |  | 0.34 | -0.13, 0.82 | 0.49 | -0.06, 1.04 |
|  | 10 (2020-22) | 0.57 | 0.14, 0.99 |  | 0.42 | -0.06, 0.91 | 0.71 | 0.01, 1.4 |

*CI:* confidence intervals.
