# Supplementary material for: Estimating the prevalence and characteristics of people in severe social isolation in 29 European countries: A secondary analysis of data from the European Social Survey round 9 (2018–2020)
Source: PLoS One. 2023 Sep 12;18(9):e0291341. doi: 10.1371/journal.pone.0291341 (PMC10497126; doi:10.1371/journal.pone.0291341)
Supplement: S5 Table — df: Degree of freedom, M: Mean, SE: Standard error. * p< 0.05, ** p< 0.01, *** p< 0.001. (DOCX) [file pone.0291341.s005.docx]

**S5 Table. Weighted descriptive statistics of sample characteristics and tests for differences’ results according to the absence/presence of severe social isolation.**

| **Variable** | **General population**  **% (95% CI)** | **Severe social isolation**  **% (95% CI)** | **F (numerator df, denominator df)- / t-value** |
| --- | --- | --- | --- |
| **Sociodemographic characteristics** |  |  |  |
| Male | 49.86 (49.02, 50.70) | 38.69 (32.41, 44.96) | 12.02 (1, 16876) *** |
| Age *M (SE)* | 40.46 (0.14) | 50.75 (0.63) | 15.71 *** |
| *Age group* |  |  | 57.82 (2, 16875) *** |
| 15-29 | - | 0.36 (0.2, 0.52) |  |
| 30-49 | - | 1.38 (1.05, 1.7) |  |
| 50-64 | - | 3.41 (2.86, 3.96) |  |
| N members household *M (SE)* | 3.04 (0.01) | 2.63 (0.10) | - 4.33 *** |
| Living alone | 12.41 (11.82, 13) | 24.42 (18.42, 30.43) | 13.64 (1, 16876) *** |
| Living with partner | 59.55 (58.67, 60.44) | 57.46 (50.98, 63.94) | 0.39 (1, 16876) |
| Living with parent | 22 (21.2, 22.79) | 8.99 (5.79, 12.2) | 43 (1, 16876) *** |
| Having children | 58.67 (57.75, 59.58) | 73.48 (67.72, 79.24) | 24.31 (1, 16876) *** |
| Living area *M (SE)* | 2.92 (0.02) | 2.89 (0.08) | - 0.44 |
| *European region* |  |  | 19.53 (3, 16874) *** |
| Northern | - | 0.83 (0.61, 1.05) |  |
| Southern | - | 1.5 (1.07, 1.93) |  |
| Western | - | 1.68 (1.3, 2.05) |  |
| Central and Eastern Europe | - | 2.84 (2.34, 3.34) |  |
| Level of education *M (SE)* | 3.99 (0.02) | 2.96 (0.12) | - 8.49 *** |
| Years of education *M (SE)* | 13.66 (0.04) | 11.42 (0.23) | - 9.53 *** |
| Disability | 3.11 (2.8, 3.43) | 32 (25.74, 38.25) | 55.36 (1, 16879) *** |
| Impairment in daily activities | 18.17 (17.47, 18.87) | 51.11 (44.64, 57.59) | 69.31 (1, 16876) *** |
| Housework | 15.94 (15.16, 16.71) | 31.57 (26.05, 37.09) | 28.77 (1, 16876) *** |
| Retired | 4.69 (4.35, 5.04) | 23.65 (18.99, 28.31) | 55.4 (1, 16876) *** |
| Internet use *M (SE)* | 4.5 (0.01) | 3.21 (0.13) | - 10.34 *** |
| Born in other country | 13.33 (12.57, 14.1) | 18.58 (12.96, 24.2) | 3.14 (1, 16876) |
| Father/Mother born in other country | 20.66 (19.78, 21.53) | 22.47 (16.66, 28.28) | 0.36 (1, 16876) |
| Father level of education *M (SE)* | 3.06 (0.02) | 2.23 (0.12) | - 6.67 *** |
| *Father working condition at 14* |  |  | 3.16 (2, 16875) * |
| Employed | 90.24 (89.68, 90.79) | 83.91 (78.91, 88.91) |  |
| Unemployed | 4.48 (4.08, 4.89) | 6.45 (3.11, 9.79) |  |
| Dead/absent | 5.28 (4.88, 5.69) | 9.64 (5.69, 13.59) |  |
| Mother level of education *M (SD)* | 2.87 (0.02) | 2.1 (0.11) | - 6.76 *** |
| *Mother working condition at 14* |  |  | 6.28 (2, 16875) ** |
| Employed | 61.22 (60.32, 62.13) | 48.59 (41.77, 55.41) |  |
| Unemployed | 37.28 (36.37, 38.18) | 48.25 (41.36, 55.13) |  |
| Dead/absent | 1.5 (1.29, 1.71) | 3.16 (0.97, 5.35) |  |
| *Household source of income* |  |  | 15.51 (3, 16874) *** |
| Wages, salaries or pensions | 92.41 (91.87, 92.95) | 63.42 (56.92, 69.92) |  |
| Unemployment/redundancy benefit | 2.27 (1.94, 2.59) | 8.28 (3.76, 12.8) |  |
| Social benefits or grants | 3.03 (2.7, 3.36) | 22.64 (16.58, 28.69) |  |
| Income from investments, savings, or other sources | 2.3 (2.02, 2.57) | 5.67 (2.6, 8.73) |  |
| Household net income (deciles) *M (SE)* | 5.86 (0.03) | 3.26 (0.17) | - 15.4 *** |
| Living uncomfortably on household income *M (SE)* | 1.87 (0.01) | 2.71 (0.07) | 12.66 *** |
| *Personal income* |  |  | 21.55 (4, 16873) *** |
| Wages, salaries or pensions | 77.69 (76.91, 78.48) | 36.08 (30.24, 41.92) |  |
| Unemployment/redundancy benefit | 2.83 (24.77, 3.18) | 8.46 (3.89, 13.02) |  |
| Social benefits or grants | 4.51 (4.13, 4.9) | 32.63 (26.01, 39.25) |  |
| Income from investments, savings, or other sources | 3.42 (3.07, 3.77) | 7.52 (3.98, 11.06) |  |
| No income | 11.54 (10.94, 12.15) | 15.31 (11.13, 19.49) |  |
| **Adverse events and fear** |  |  |  |
| Burglary or assault | 17.25 (16.55, 17.95) | 11.38 (7.14, 15.62) | 6.95 (1, 16876) ** |
| Feeling unsafe when walking alone *M (SE)* | 1.93 (0.01) | 2.3 (0.06) | 6.12 *** |
| **Emotional social support and trust** |  |  |  |
| Emotional support *M (SE)* | 3.05 (0.02) | 1.57 (0.08) | - 18.29 *** |
| Social trust *M (SE)* | 5.22 (0.02) | 3.71 (0.13) | - 11.82 *** |
| Political trust *M (SE)* | 3.39 (0.01) | 2.6 (0.1) | - 7.43 *** |
| **Well-being** |  |  |  |
| Satisfaction with life *M (SE)* | 7.24 (0.02) | 4.82 (0.18) | - 13.39 *** |
| Happiness *M (SE)* | 7.6 (0.02) | 5.42 (0.18) | - 12.37 *** |
| Bad general health *M (SE)* | 2.04 (0.01) | 2.99 (0.08) | 12.61 *** |
| Lack of planning for the future *M (SE)* | 4.88 (0.03) | 6.58 (0.2) | 8.48 *** |

*df:* degree of freedom, *M:* mean, *SE:* standard error.

* *p*< 0.05, ** *p*< 0.01, *** *p*< 0.001.
