# Supplementary material for: Estimating the prevalence and characteristics of people in severe social isolation in 29 European countries: A secondary analysis of data from the European Social Survey round 9 (2018–2020)
Source: PLoS One. 2023 Sep 12;18(9):e0291341. doi: 10.1371/journal.pone.0291341 (PMC10497126; doi:10.1371/journal.pone.0291341)
Supplement: S6 Table — df: Degree of freedom. * p< 0.05, ** p< 0.01, *** p< 0.001. (DOCX) [file pone.0291341.s006.docx]

**S6 Table. Weighted descriptive statistics of indicators of severe social isolation according to the European region.**

| **Variable** | **General population**  **% (95% CI)** | **F (numerator df, denominator df) -value** |
| --- | --- | --- |
| **Social meeting with friends, relatives or colleagues less than once a month or never** |  |  |
| *European region* |  | 77.25 (3, 16874) *** |
| Northern | 4.48 (3.94, 5.02) |  |
| Southern | 6.83 (5.89, 7.77) |  |
| Western | 6.28 (5.62, 6.95) |  |
| Central and Eastern Europe | 14.11 (12.98, 15.23) |  |
| **Taking part in social activities less than most or much less than most compared to others of same age** |  |  |
| *European region* |  | 28.6 (3, 16874) *** |
| Northern | 35.32 (33.9, 36.74) |  |
| Southern | 28.02 (26.43, 29.62) |  |
| Western | 37.76 (36.41, 39.11) |  |
| Central and Eastern Europe | 33.81 (32.29, 35.33) |  |
| **Not working (or not away temporarily) during the last week** |  |  |
| *European region* |  | 33.73 (3, 16874) *** |
| Northern | 28.18 (26.79, 29.58) |  |
| Southern | 39.01 (37.24, 40.78) |  |
| Western | 30.1 (28.81, 31.4) |  |
| Central and Eastern Europe | 33.51 (32.09, 34.92) |  |
| **Not actively looking for a job during the last week** |  |  |
| *European region* |  | 40.34 (3, 16874) *** |
| Northern | 96.84 (96.38, 97.3) |  |
| Southern | 90.6 (89.59, 91.6) |  |
| Western | 96.08 (95.49, 96.68) |  |
| Central and Eastern Europe | 95.93 (95.38, 96.48) |  |
| **Not being in education (not paid for by employer), even if on vacation, during the last week** |  |  |
| *European region* |  | 24.03 (3, 16874) *** |
| Northern | 80.23 (78.91, 81.54) |  |
| Southern | 85.37 (84.12, 86.62) |  |
| Western | 86.57 (85.61, 87.54) |  |
| Central and Eastern Europe | 86.99 (85.96, 88.01) |  |

*df:* degree of freedom.

* *p*< 0.05, ** *p*< 0.01, *** *p*< 0.001.
