# Supplementary material for: Estimating the prevalence and characteristics of people in severe social isolation in 29 European countries: A secondary analysis of data from the European Social Survey round 9 (2018–2020)
Source: PLoS One. 2023 Sep 12;18(9):e0291341. doi: 10.1371/journal.pone.0291341 (PMC10497126; doi:10.1371/journal.pone.0291341)
Supplement: Attachment — Submitted filename: Response to Reviewers.docx [file pone.0291341.s007.docx]

**Reviewer#1’s comments**

The article’s main aim is to estimate high social isolation in Europe and posit its relevance for hikikomori. In addition, it delves into the factors that predict high social isolation.

Operationalization: the definition of hikikomori includes also the subjective feeling of isolation (e.g. the feeling of ”distress”, but this subjective dimension is not included in the authors’ measure of extreme isolation. The reason for and implications of leaving out this ”loneliness” or other type of mental subjective consequences of social isolation need to be discussed, since this is an essential feature of hikikomori. Also the indicators used to measure social isolation here (few social meetings, few social activities, not working, not job seeking, not in education); the readers do not see how these are put together into an index. Is it an additive index or constructed from factors? Or is it a multiplicative index, because hikimori symptoms should be found in combination? This is crucial to understand the claim. With this information (and the subjective dimension) missing, I find it hard to go along with the central claim, that this measure of extreme social isolation is a proxy for hikikomori. This paper is actually about extreme social isolation in Europe. I find the hikikomori aspect of this both distracting, not supported, and perhaps less theoretically interesting than the more general issue of extreme social isolation. What additionally is gained by the ”hikikomori” concept in a European context?

**Authors’ reply**: Dear Reviewer#1, thank you for your attention in reviewing our manuscript and for your valuable comments.

We agree with you on the importance of studying significant impairment or distress associated with social isolation as a core criterion of hikikomori. However, this should be also contextualized considering current research as well as the opportunity to study the phenomenon or a good proxy of it such as severe social isolation. Therefore, we have specified that “*Further, these two studies as well as forty-six (88.5%) out of fifty-two studies analysed in the systematic review mentioned above (14) did not consider significant impairment or distress associated with marked isolation as an inclusion criterion for the definition of hikikomori.*” (lines 83-86) in the introduction section of the new version of the manuscript when discussing hikikomori definitions. One may thus wonder whether all those studies examined something different from hikikomori such as a condition at risk of hikikomori or severe social isolation. Additionally, we included the following among the study limitations “*Further, significant impairment or distress caused by social isolation was not included in the definition of severe social isolation in line with the fact that we examined a condition at high risk for hikikomori rather than hikikomori itself. It should be noted that most studies (88.5%) examining hikikomori, in reality, did not consider significant impairment or distress (14) and, thus, the validity of their findings is questionable.* *The inclusion of specific psychological indicators of dysfunction and impairment/distress caused by social withdrawal and isolation is needed in future studies of hikikomori (10-14).” (lines 542-548*). Regarding the indicators used to measure severe social isolation in the present study, we specified that “*If all five indicators were present, an individual was classified as in severe social isolation*.” (line 145) under the paragraph “Measures - Severe social isolation as a proxy for high risk of hikikomori” in the new version of the manuscript. Considering that our definition of severe social isolation as a proxy for high risk of hikikomori focused on both the lack of social participation and relationships (in line with initial and more conservative definitions of hikikomori such as Teo and Gaw 2010), we examined a more severe/extreme condition rather than social isolation (i.e., usually defined as few/lack of social contact) itself. Accordingly, we specified that “*Therefore, the condition examined differs from social isolation in general, as evaluated considering objective indicators of frequency and/or number of social contacts. The inclusion of lack of social participation (not working and not searching for work, not being in education) and social relationships as defined above - but not significant impairment and/or distress - (13, 14) enabled us to examine severe social isolation as a proxy for high risk of hikikomori.*” (lines 146-150). Therefore, another added value of our study, as discussed in the discussion section, is the fact that we question the validity of the most recent definition of hikikomori (Kato et al., 2019, 2020) because it does not clarify the differences between hikikomori and severe social isolation (indeed, people in severe social isolation may spend an increased amount of time at home and experience significant distress caused by social isolation).

**Reviewer#1’s comments:** Theory: If this were an example of hikikomori, if this operationalization were convincing, what can we do with these findings? We learn that hikomori is also present in Europe, we learn about some of determinants, but we do not know why, theoretically, its prevalence varies. For example, there is the evidence that this isolation is more widespread in Central and Eastern Europe, but we do not get a discussion about why. There is growing social-scientific research about why social isolation and loneliness vary culturally, but none of this work is cited. As such, we get a list of independent variables and their impacts, but readers are left to guess why they are included and how to interpret their findings. My advice would be to make use of sociological, cultural, and comparative social-psychological findings on these themes to try to disentangle where enhanced social isolation is found in Europe and why.

**Authors’ reply**: Thank you for your helpful suggestion. Please note that we included - already in the first version of the manuscript - a specific section (“Implications for hikikomori research”) to clarify the implications of the findings of our study for hikikomori research. Considering that the discussion was dense enough, we missed paying sufficient attention to explaining the higher rate of severe social isolation in Central and Eastern Europe compared to the Northern part in the first version of the manuscript. Further, the main aim of the present study was not to specifically analyse differences between countries/European regions. Indeed, it was to estimate the overall prevalence and analyse demographic and psychosocial variables with the European region being only one of them.

Despite the above, in accordance with your suggestion, we investigated the weighted prevalence of each indicator used for severe social isolation across European regions (results in Table S5) to provide additional information - as well as useful scientific references (see below) - to interested readers. The following text was added to the discussion: “*We found that Central and Eastern Europe differed from Northern Europe in the prevalence of three out of five indicators of severe social isolation (Table S5). Specifically, the prevalence of social meeting with friends, relatives, or colleagues less than once a month or never, not working, and not being in education, was higher in Central and Eastern Europe than in Northern Europe. This point thus to the potential role of socio-economic country-level factors that make individual residents more at risk of severe social isolation as previously hypothesized about loneliness (47-49).* *It should be considered that much more previous research analysed differences in loneliness rather than in social isolation across European countries. And severe social isolation was analysed especially with samples of older adults rather than with the working-age population. Future research may benefit from investigating the potential role of country-level factors such as gross domestic product per capita, social inequality, poverty, social expenditure, and/or education spending (see, for example, the following useful references about the analysis of macro-determinants of NEET and loneliness 50–55) on the prevalence of severe social isolation.* *Critically, studies aiming at examining between countries’ similarities and differences in risk and protective factors of severe social isolation and hikikomori could be conducted*.” (lines 364-378).

In light of the above, especially the lack of studies on severe social isolation in the working-age population, and for avoiding creating confusion between social isolation and loneliness (in accordance with comments of Reviewer#2), we underscored the importance of future studies on macroeconomic determinants (as previously done for studying NEET and loneliness) of severe social isolation.

**Reviewer#1’s comments:** Likewise, I was not convinced by the brief trend analysis, going against a growing vast research, that suggests social isolation did not increase during COVID years. An analysis of this type would really have to take subgroups into account, rather than country-level averages, to look at social isolation changes within subgroups.

**Authors’ reply**: Thank you for your comment. Considering your valuable suggestion, we added a new paragraph to the results section and conducted additional analysis.

Please note that we analysed severe social isolation rather than social isolation in general. Therefore, as we highlighted in the first version of the manuscript (discussion section), “Finally, our findings differ from the decline in social connectedness observed in the USA (46) likely because of the different operationalization of isolation (i.e., severe social isolation versus time spent with others).”. At the same time, it should be noted that the quoted study from the USA showed a decreasing trend in social connectedness that started well before the COVID-19 pandemic. While d’Hombres et al. (<http://hdl.handle.net/10419/236276>) showed that social isolation remained relatively stable, despite a slightly increasing trend, between 2002 and 2018in European countries overall.

Further, we agree that the social distancing measures during the COVID-19 pandemic clearly exerted an impact on objective measures of social isolation in general, but with this effect being temporary and/or affecting mainly adolescents and young adults. The effect of social distancing measures on severe social isolation could have been negligible. Of importance, the working-age population could have been at risk not due to social distancing but rather due to subsequent economic downturn. In line with this, we highlighted that “However, future studies are needed to test whether changes in economic stability and inequality could, in turn, influence the prevalence of severe social isolation and hikikomori.” in the first version of the manuscript.

However, we expanded the trend analysis, in accordance with your comment, analysing the trend of severe social isolation for each of the ten countries with available data for the three ESS rounds. The following text was added to the results section of the new version of the manuscript under the new paragraph “Prevalence and trend of severe social isolation by country”: “*As recommended during the review process, we also examined trends in severe social isolation according to the ten countries with available data for each of the three ESS rounds to further inform the trend analysis (S4 Table). The decrease in total prevalence over time was significant (i.e., confidence intervals do not overlap) for data from Hungary and Slovenia. On the contrary, no other significant trend was found despite overall decreasing (for the Czech Republic, France, Italy, and the Netherlands) and increasing (for Finland, Norway, Portugal, and Switzerland) trends. The data at the country level, especially by sex, should be interpreted with caution considering the low prevalence and wide confidence intervals. The relatively small samples size at the country level (sample size ranges from 919 (Czech Republic) to 1,916 (Netherlands) for ESS 1, from 736 (Portugal) to 1,938 (Italy) for ESS 9, and from 935 (Slovenia) to 1,930 (Czech Republic) for ESS 10) may have decreased statistical power*.” (lines 229-241).

The following was added to the discussion: “*Similarly, research with representative samples should confirm these findings at the country level of analysis*.” (line 344).

Kind regards,

The authors

**Reviewer#2’s comments**

Thank you for the opportunity to review this manuscript “Estimating the prevalence and characteristics of people in severe social isolation in 29 European countries: A secondary analysis of data from the European Social Survey round 9 (2018-2020)”. This study thoughtfully estimates the prevalence of people in severe social isolation as a proxy for high risk of hikikomori using data from 29 European countries. Additionally, it considers the impacts of relevant psychological and social variables on social isolation.

**Authors’ reply**: Dear Reviewer#2 thank you for your time in reviewing our manuscript and for your positive comment overall. Please find below our replies to your helpful comments/suggestions.

**Reviewer#2’s comment:** Line 49-51. Social isolation is defined and references as a objective and subjective construct. Consider revising this text as much of current think considers social isolation to be an objective state and strives to distinguish this from loneliness. This is described in the recent NASEM report on social isolation and loneliness as well as Age UK information- https://www.ageuk.org.uk/our-impact/policy-research/loneliness-research-and-resources/loneliness-isolation-understanding-the-difference-why-it-matters/.

**Authors’ reply**: Thank you for this comment (and the online reference) that gives us the opportunity to clarify what social isolation is according to the quoted references (Wang et al., 2017). We agree that such a differentiation is extremely relevant. In accordance with the authors, we added that social isolation refers to “*inadequate quality and quantity of social relations.* *In accordance with the authors (5), social isolation contains a subjective judgement besides a more objective one while loneliness is entirely subjective*” (lines 51-51). This sentence was added to the text in the new version of the manuscript to further clarify the meaning of “subjective perceived adequacy of contact”. For instance, having friends to count on in difficult moments is a personal evaluation of the “subjective perceived adequacy of contact”. It is still in the realm of social isolation because related to the number of friends, but it involves a subjective evaluation (on how many friends, if any, ones can count on in difficult moments). The two aspects may not overlap. However, they differ from loneliness, which is generally conceptualized as dissatisfaction with the discrepancy between desired and actual social relationships (Peplau & Perlman, 1982). Therefore, we would prefer to maintain the definition as it was.

**Reviewer#2’s comment:** Line 164- 166. Authors have opportunity to be more specific that this is referring to emotional support rather than broader domain of social support based on the question that is included in the study.

**Authors’ reply**: Thank you for your helpful comment. In accordance, we have specified “emotional” support throughout the manuscript.

**Reviewer#2’s comment:** Line 343-344. Authors make a loneliness reference however this conceptually differs from social isolation.

**Authors’ reply**: Thank you for your comment and kind attention. In the new version of the manuscript, the sentence was removed from the text to avoid confusion.

Kind regards,

The authors
